# Infective Endocarditis Caused By Streptococcus pluranimalium: A Case Report

**DOI:** 10.7759/cureus.49219

**Published:** 2023-11-22

**Authors:** Fahad S Alqahtani, Hiba Al-Asadi, Abdulaziz I Alsenani, Wejdan S Alanazi, Abdulaziz G Alanazi

**Affiliations:** 1 Internal Medicine, Prince Sultan Military Medical City, Riyadh, SAU; 2 Infectious Disease, Specialized Medical Center Hospital, Riyadh, SAU; 3 Internal Medicine, Specialized Medical Center Hospital, Riyadh, SAU

**Keywords:** septicemia, infectious disease, streptococcus pluranimalium, cardiology, infective endocarditis

## Abstract

Infective endocarditis (IE) refers to an infection of the endocardium that can be caused by bacteria, viruses, or fungi. It can manifest either acutely or sub-acutely. Which can be complicated to stroke and congestive heart failure. We report a case of IE caused by Streptococcus (S.) pluranimalium. It is a rare organism that was discovered in 1999 in infections affecting domestic animals. It can cause serious complications, such as brain abscesses, and IE in both humans and animals. Our patient was diagnosed with IE caused by S. pluranimalium based on modified Duke’s criteria. After the diagnosis was made, the patient started on intravenous antibiotics according to the blood cultures. Then, the patient significantly improved. We are reporting this case because only a few cases were documented for infective endocarditis caused by S. pluranimalium.

## Introduction

Infective endocarditis (IE) refers to an infection of the endocardium that can be caused by bacteria, viruses, or fungi. It can manifest either acutely or sub-acutely [1]. Each year, between 1.4 and 12.7 cases are reported for every 100,000 individuals [2]. The mortality rate can be as high as 30% within 30 days [3,4]. Two major and dangerous complications of IE are stroke and congestive heart failure, which are increasing mortality. Streptococcus viridans is frequently identified as the most common pathogen leading to subacute IE, while acute IE is often caused by Staphylococcus aureus [1]. Streptococcus (S.) pluranimalium is a gram‐positive bacteria [5], which was discovered by Devriese et al. (1999) in infections affecting domestic animals [5,6]. Currently, human infections caused by S. pluranimalium are rare but documented. They can cause IE, septicemia, and brain abscesses [5]. In our case, we reported a young lady presented with high-grade fever and found that she had bacteremia caused by S. pluranimalium complicated by IE.

## Case presentation

**Figure 1:**
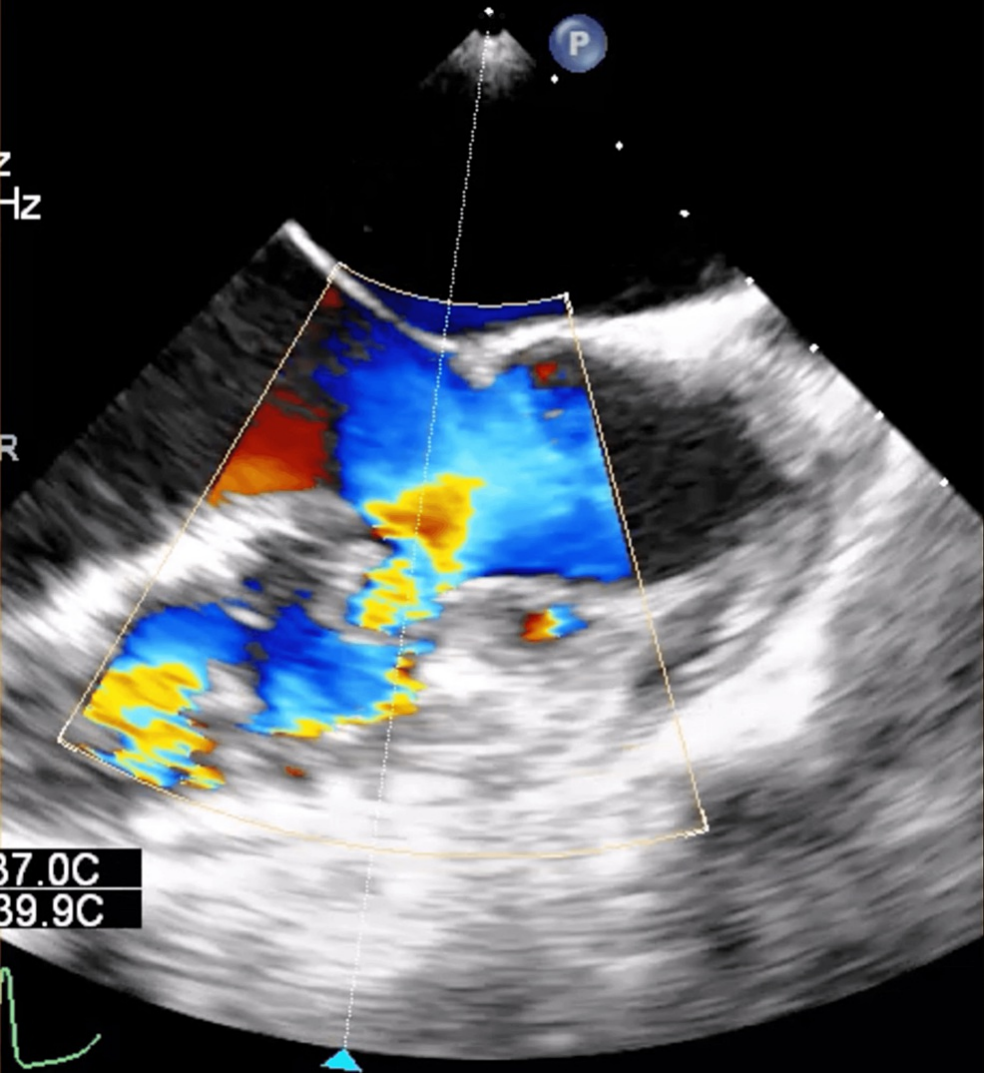
Color Doppler mid-transesophageal echocardiography showing a ventricular septal defect (VSD) from the left to right ventricular side

**Figure 2:**
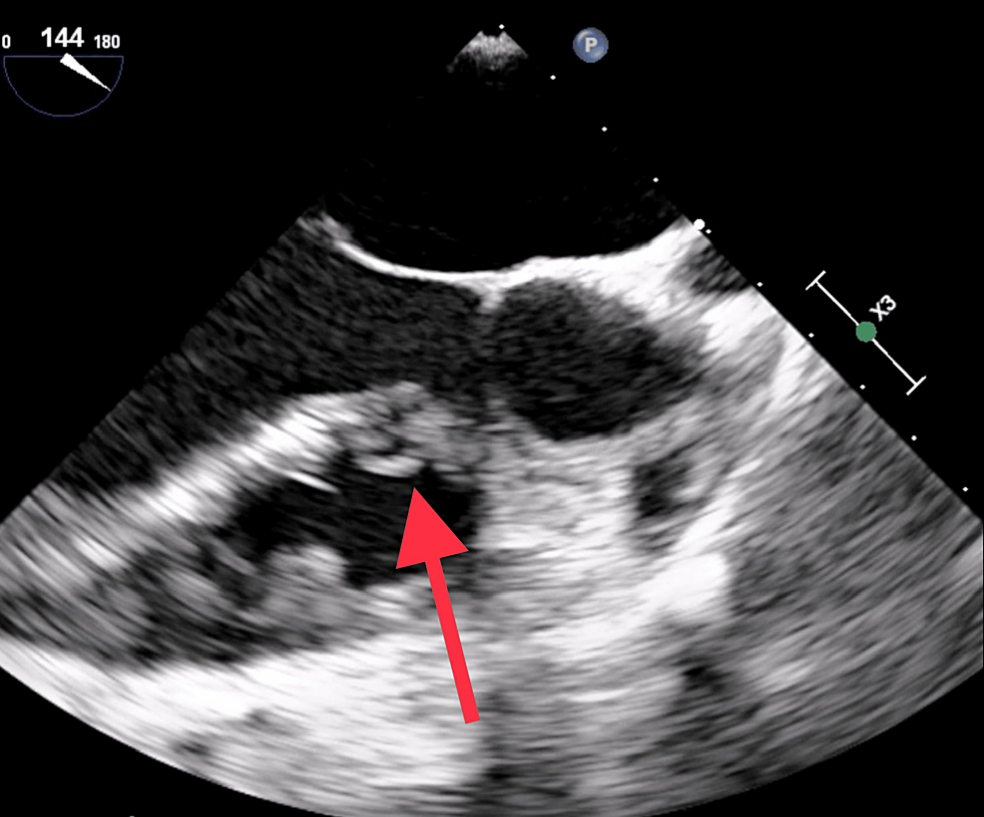
Transesophageal echocardiography view at angle 144 showing a mass attached to the septal wall from the right ventricular side, representing vegetation

A 28-year-old Pakistani female without a past medical history of significant diseases visited the infectious disease clinic, complaining of fever. The fever started three weeks before visiting the hospital as it reached 39 °C. The patient did not have any other symptoms, such as shortness of breath, coughing, chest pain, skin rash, joint pain, abdominal pain, dysuria, hematuria, bowel habits changes, headache, and neurological symptoms. Also, the patient denied any recent dental procedures, contact with animals, using illicit intravenous drugs, and smoking. The patient's vitals were blood pressure: 107/72 mmHg, oxygen saturation 98% in room air, heart rate: 95 beats per minute, respiratory rate: 20 breaths per minute, and temperature: 39 °C. Physical exam was unremarkable except for cardiovascular auscultation harsh pansystolic murmur. An initial workup was done (Table 1), and two sets of blood cultures were obtained and showed gram-positive Cocci for two bottles, and the chest X-ray was unremarkable. Then, the patient was admitted for intravenous antibiotics and an echocardiogram for IE as part of the workup. The patient started on vancomycin. Two sets of blood cultures showed S. pluranimalium and were sensitive to vancomycin, ceftriaxone, and amoxicillin/clavulanic acid. Transthoracic echocardiogram showed there is a perimembranous ventricular septal defect (VSD), left ventricular ejection fraction of 55-60%, no regional wall motion abnormalities, and no vegetation or masses. Transesophageal echocardiography was performed and showed a membranous ventricular septal defect of moderate size (Figure 1). There is a small mobile mass attached to the septal wall on the right ventricular side at the jet path of VSD likely representing vegetation (Figure 2). Further tests were performed on the patient including autoimmune and HIV, which showed negative results. The patient was diagnosed with IE caused by S. pluranimalium based on modified Duke’s criteria that the patient fulfilled: one major criterion (intracardiac vegetation) and three minor criteria (congenital heart disease, fever, and positive blood culture). The intravenous antibiotics were de-escalated to ceftriaxone for a total of four weeks, then the fever subsided and the patient showed clinical improvement. After that, she was referred to cardiology for the VSD.

**Table 1:** Laboratory findings

investigation	result
White blood cell count	9.09 K/UL
Hemoglobin	12.40 g/dl
Platelet count	229 10S3/UL
ESR	95.000 mm/hr
CRP	1.1 mg/dl
Creatinine	0.85 mg/dl
Urinalysis	Unremarkable
Brucella abortus antibody (latex agglutination)	1:80
Brucella melitensis antibody (latex agglutination)	1:80

## Discussion

IE occurs when the endocardial surface of the heart becomes infected, and the diagnosis for our patient was established based on modified Duke’s criteria that the patient fulfilled. Sometimes, blood culture can be negative in some situations, for example, if the patient used previous antibiotics, or for some other organisms like the HACEK group (Haemophilus species, Actinobacillus actinomycetemcomitans, Cardiobacterium hominis, Eikenella corrodens, and Kingella kingae), Legionella, Histoplasma, Aspergillus, and Candida [1]. There are some predisposing factors for IE like patent ductus arteriosus, ventricular septal defect, valvular heart disease, and the use of illicit intravenous drugs [2,6]; our patient had one of them. Approximately 80% to 90% of IE cases can be attributed to infections caused by Staphylococci, Streptococci, and Enterococci while Staphylococcus aureus is identified as the primary microorganism in the case of right-sided IE for intravenous drug users. Streptococcus viridans is also identified in non-drug users [2]. S. pluranimalium is a rare type of Streptococcus that has been discovered and documented in cases of infections in both humans and animals. Studies have shown that a reliable treatment plan for IE and septicemia infections would be using a combination of vancomycin, tetracycline, and third-generation cephalosporins [5]. In cases where the patient's condition worsens and medical therapy proves ineffective, surgery may be required for IE. This scenario occurs in almost 50% of the cases, especially when the patient develops symptoms of heart failure, not controlling the infection; vegetation size is important as well [4].

## Conclusions

IE refers to an infection of the endocardium that can be caused by many organisms. It can cause stroke and congestive heart failure as complications. The patient should fulfill the modified Duke’s criteria for the diagnosis (two major, or one major and three minor). The case can be managed medically, but if the patient does not respond to the treatment, surgical interference for IE will be required. The unusual presentation in our case is the organism, S. pluranimalium. The patient initially received intravenous vancomycin, which was de-escalated after the result of the blood culture to intravenous ceftriaxone for a total of four weeks. As a result, the patient showed clinical and radiological improvement. Therefore, it is important to know that S. pluranimalium can cause bacteremia in humans that may cause endocarditis or brain abscesses even without solid evidence of contact with animals.
